# TRPV4 mediates afferent pathways in the urinary bladder. A spinal c-fos study showing TRPV1 related adaptations in the TRPV4 knockout mouse

**DOI:** 10.1007/s00424-016-1859-9

**Published:** 2016-08-05

**Authors:** Dick A. W. Janssen, Joost G. Hoenderop, John P. F. A. Heesakkers, Jack A. Schalken

**Affiliations:** 1 Department of Urology, Radboud University Nijmegen Medical Center, Geert Grooteplein 10, 6500 HB Nijmegen, The Netherlands; 2 Department of Physiology, Radboud University Nijmegen Medical Center, Nijmegen, The Netherlands

**Keywords:** TRPV4, Urothelium, C-fos, Bladder, C-fiber, Afferent signaling

## Abstract

The role of transient receptor potential vanilloid subtype 4 (TRPV4) channels in urinary bladder afferent neural pathways was investigated using spinal c-fos measurements in mice. Anesthetized wild type and TRPV4 knockout (−/−) mice underwent noxious bladder distention and treatment with either intravesical instillation with lipopolysaccharide (LPS), or the TRPV1 agonist resiniferatoxin (RTX), vehicle or an intraperitoneal injected TRPV4 antagonist (HC067047). Mice underwent paraformaldehyde perfusion for rapid fixation and L6-S1 spinal cord sections were removed followed by immunohistochemical staining for c-fos. A number of c-fos expressing neurons in the dorsal horns of L6-S1 spinal cord transections were quantified. Groups were compared using univariate ANOVA. Even with the absence of bladder inflammation on H&E, the TRPV4 −/− mice still have a significant twofold higher c-fos expression (*n* = 39, SD 2) after noxious bladder distention compared to wild type mice (*n* = 20, SD 3). A twofold increase in c-fos expression was observed after LPS treatment in wild types (*n* = 42, SD 5), but no increase was seen in TRPV4 −/− mice (*n* = 42, SD 2). After desensitization of primary afferent C-nerve fibers with RTX, c-fos expression in TRPV4−/− mice decreased significantly (threefold) (*n* = 12, SD 4). Results imply that TRPV4 channels are important for bladder afferent signaling. TRPV4 −/− mice bladders generate more noxious sensory output, which is predominantly mediated through TRPV1 expressing high threshold nerve fibers. This study reveals TRPV1 related adaptive changes in afferent pathways of the TRPV4 −/− mouse. We propose that this effect is caused by a congenital impairment of low threshold nerves that mediate normal bladder filling sensations.

**Electronic supplementary material:**

The online version of this article (doi:10.1007/s00424-016-1859-9) contains supplementary material, which is available to authorized users.

## Introduction

Transient receptor potential vanilloid subtype 4 (TRPV4) is a non-selective Ca^2^^+^-permeable cation channel that is located on the membranes of urinary bladder epithelial cells [9, 14, 19, 20]. TRPV4 is part of the large family of TRP-channels that are involved in a wide range of physiological processes and is the most abundantly expressed TRP-channel in the urinary bladder [8, 9, 21]. TRPV4 channels are of interest because the channel is activated by mechanical stretch and therefore could participate in bladder filling sensory pathways [8, 11, 17, 24].

Under physiological conditions, stretch in the urothelium causes a TRPV4 mediated Ca^2^^+^ influx into the cell which triggers the release of ATP, a potent activator of afferent nerve fibers in the bladder [8, 11, 17, 24]. These afferents contain low and high threshold nerve fibers, that mostly consist of myelinated Aδ fibers, and a group of silent fibers that consist of unmyelinated C-nerve fibers [11, 15, 17, 28]. Low threshold fibers are important for normal bladder filling sensations and respond to low bladder pressures. High threshold fibers are only activated during high pressures (>20 cmH2O) and silent fibers are activated during chemical irritation after which they also become mechanosensitive. The high threshold fibers express TRPV1 receptors and are important for inflammatory responses and pain [28].

A considerable number of publications suggest that TRPV4 channels are involved in bladder afferent pathways [3, 10, 11, 17]. The phenotype of transgenic TRPV4 −/− mice is marked by an enlarged bladder capacity, diminished voiding contractions and a reduced ATP response to urothelial stretch [11, 17]. This body of evidence implies that TRPV4 −/− mice have inadequate bladder filling sensations which mostly occurs through low threshold afferents [28].

These characteristics make TRPV4 channels an interesting target for pharmacological intervention for conditions like overactive bladder syndrome (OAB) or bladder pain syndrome (BPS). Evidence from in vitro and in vivo experiments confirm that the pharmacological blocking and stimulating of TRPV4 channels can mediate a local response in the bladder [3, 10, 25, 26]. Investigating the sensory function of TRPV4 channels higher up in the afferent neural tract could give more insight in the function of these channels in the lower urinary tract. To gain this insight, neural sensory information needs to be quantified.

The spinal c-fos experimental model is an in vivo model that enables us to quantify afferent signaling [3, 5, 13]. After stimulation, c-fos mRNA is transcribed in the nuclei of sensory neurons [7, 18]. In mice, the afferent signals that originate in the bladder travel via the dorsal root ganglia and enters the spinal cord at the superficial dorsal horn area of the spinal cord segment L6-S1 [1, 2]. Noxious stimuli in the bladder results in the transcription of c-fos in the three main areas of the L6-S1 segment that are important in bladder afferent signaling. These are the dorsal horns (DH) (laminea I, II, V), sacral parasympathetic nuclei (SPN), and the dorsal commissure [1, 2, 13, 27].

The aim of this study was to investigate the contribution of TRPV4 channels in bladder afferent signaling by quantifying c-fos positive nuclei in sensory regions of the L6-S1 spinal cord segment of normal wild type and transgenic TRPV4 −/− mice.

## Materials and methods

### Animal experiment

Our animal experiments were conducted with the approval of our regional animal ethical committee Radboud University Nijmegen Medical Centre (CDL). Wild type mice (C57BL/6UJ) (*n* = 16) and TRPV4 −/− (*n* = 16) females of 25 g were used for experiments. TRPV4 −/− mice (*n* = 16) were generously made available by Suzuki et al. [23]. Knock out of TRPV4 in TRPV4 −/− mice was validated by Western blotting and immunohistochemistry experiments [14]. The experimental procedure went as followed. On day 1, mice were anesthetized with isoflurane inhalation (1.5 %) and catheterized using a thin PET tube (PE10; Intramedic Sparks, USA) and gel lubrication. Bladders were emptied.

Four different experimental groups were created. (1) A *control* group (TRPV4 −/− *n* = 4, wild type *n* = 4) received a bladder installation of 0.1 ml of normal saline for 10 min. (2) A *bladder inflammation* group (TRPV4 −/− *n* = 4, wild type *n* = 4) received an installation of 0.1 ml Lipopolysaccharide (LPS, 5 mg/ml) (Sigma-Aldrich, St Louis, USA) in the bladder for 10 min. (3) A *resiniferatoxin* (RTX) group (TRPV4 −/− *n* = 4, wild type *n* = 4) received a bladder instillation of 0.1 ml RTX (1 nM dissolved in DMSO) (Tocris Bioscience, Bristol, UK) for 30 min to desensitize TRPV1 expressing high threshold and silent afferent C-fibers. (4) A *TRPV4 antagonist* group (TRPV4 −/− *n* = 4, wild type *n* = 4) received an installation of 0.1 ml of normal saline at day 1 followed by an intraperitoneal (ip) injection of the TRPV4 antagonist *HC 067047* (10 mg/kg) (Tocris Bioscience, Bristol, UK) 15 min prior to the surgical intervention at day 2.

**Fig. 1:**
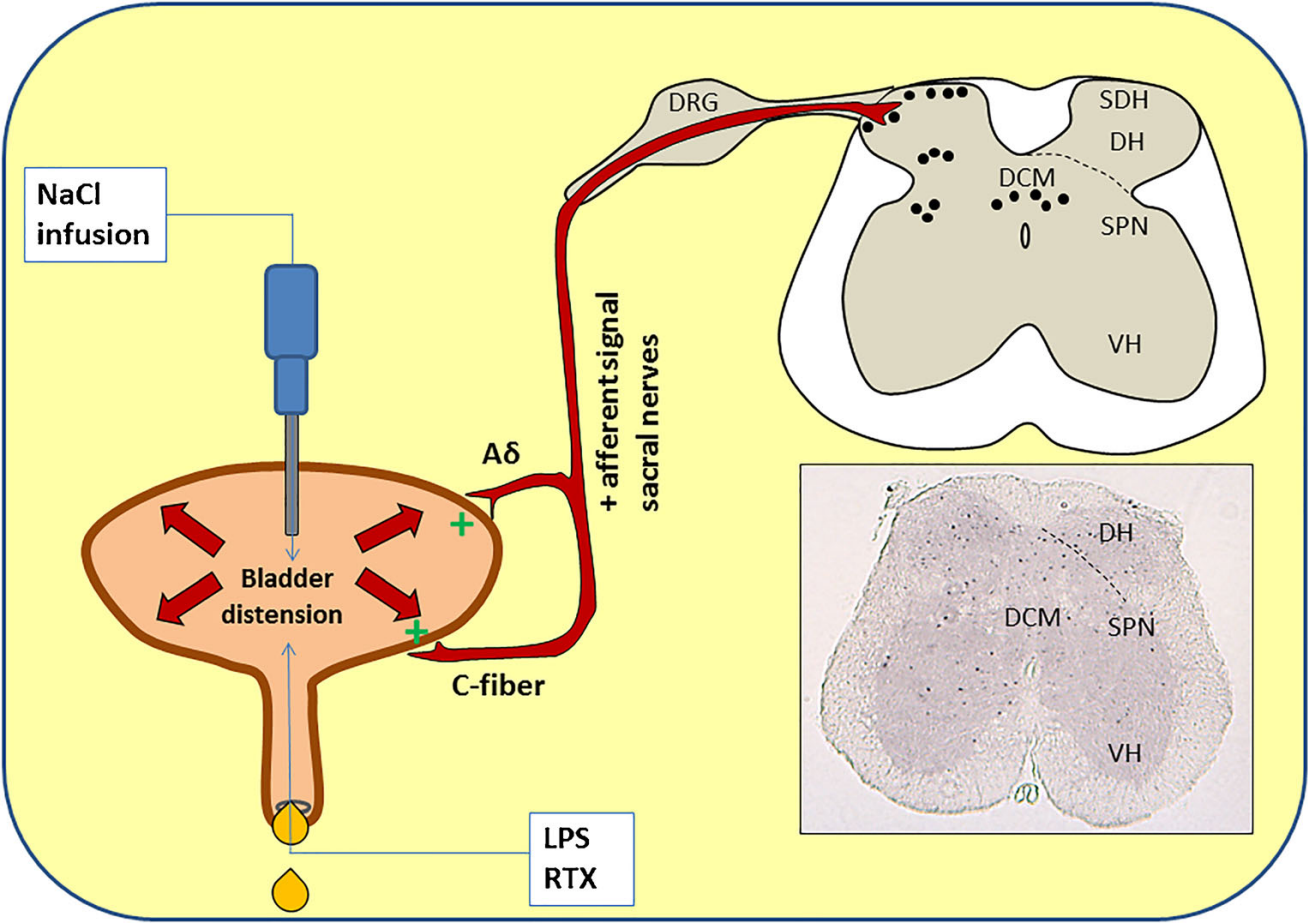
Applied experimental spinal c-fos in vivo mouse model for bladder afferent signaling. Bladder distension is achieved via normal saline infusion through a small syringe inserted into the bladder dome. LPS, RTX or vehicle was instilled in the bladder via catherisation. Bladders were able to empty during noxious distension (23 cmH2O). During stimulation, afferent nerves (Aδ and C-fibers) are activated, and afferent signal travels through the sacral nerves, cross the dorsal root ganglia (*DRG*) and enter the superficial dorsal horn (*SDH*) areas of the L6-S1 spinal cord segment, where nuclei of afferent nerves start producing c-fos in three main areas: the dorsal horn (*DH* and *SDH*), sacral parasympathetic nuclei (*SPN*), and the dorsal commissure (*DCM*). The bottom right image shows a c-fos immunostaining of a L6-S1 spinal cord transection of a TRPV4 −/− mouse that has underwent a bladder distention. *Black dots* are c-fos expressing nuclei. VH = ventral horn

At day 2 (24 h later), mice from all groups were anesthetized with urethane ip (2 g/kg). The abdomen was surgically opened and an infusion needle was inserted in the bladder dome. Bladder contractions and voiding was still possible. The bladder was then continuously infused with normal saline at 23 cmH2O (noxious distention) for 60 min (37 °C) (Fig. 1).

After this, the thorax was quickly opened. A needle was inserted first in the portal vein and secondly through the left cardiac ventricle, after which the blood supply was drained by flushing the circulatory system with phosphate buffered saline (PBS) (0.4 M pH 7.2). Immediately, after this, the mice underwent a perfusion fixation procedure by flushing the circulatory system with 4 % (*v*/*v*) paraformaldehyde in 0.1 M PBS (pH 7.2), until paws and tail stiffened up. Mice were then placed in flasks containing 4 % (*v*/*v*) paraformaldehyde in 0.1 M PBS (pH 7.2) and transported to the lab for tissue preparation.

### Tissue preparation

Spinal cords including dorsal roots ganglia were surgically removed using a dissection microscope (Zeiss, Göttingen, Germany) and microscopic surgery tweezers and cutting scissors and subsequently fixated one night in 4 % (*v*/*v*) paraformaldehyde and 24 h in a 30 % (*v*/*v*) sucrose in 0.1 M PBS solution. The L6-S1 spinal cord segment was isolated and imbedded separately with Tissue-Tek® O.C.T. (Sakura Finetek, Japan) and snap frozen in isopentane on dry ice and stored at −80 °C. Sections (35 μm thick) were cut with a freezing microtome (Microm, Heidelberg, Germany) and collected in free floating staining cups that were placed in 12 well plates (Millipore Corporation, Billerica, USA) containing 0.1 M PBS solution (pH 7,3). Rat spinal cord cross sections that had received a similar fixation protocol were used as positive controls. Also, urinary bladders were removed and immediately snap frozen in isopentane on dry ice and cut in 4-μm sections with a freezing microtome and placed on glass slides (Superfrost®plus, Thermo-Scientific, Waltham, USA).

### Immunohistochemistry

Bladder tissue was analyzed for signs of inflammation with standard HE staining. Free floating immunohistochemistry was performed for spinal cord c-fos experiments. Tissue was rinsed with 0.1 M PBS solution containing 0.1 % (*v*/*v*) bovine serum albumin and 0.3 % (*v*/*v*) Triton-X-100 (PBS-BT). Tissue was incubated overnight with a c-fos (4) antibody (c-fos (4) sc-52 rabbit-polyclonal Lot # L 1809, Santa Cruz®, Santa Cruz, USA) dissolved in PBS-BT at room temperature. We used 0.1 M PBS pH 7.3 for further rinsing steps. Tissue was incubated with a donkey-a-rabbit IgG biotin conjugated secondary antibody (Jackson Imm Research®, West Grove, USA) in PBS-BT for 90 min, following incubation with Vector ABC elite® (Vector, Burlingame, USA) for 90 min. Tissue was incubated using a DAB solution containing 10 μl DAB (Sigma-Aldrich, St Louis, USA) and 150 mg ammonium-nickel-sulfate dissolved in 50 ml 0.05 M Tris buffered saline for 10 min, followed by a 10 min incubation with the same DAB-nickel solution with the addition of perhydrol (5 μl in 25 ml DAB-nickel solution). Tissue was mounted on objects glasses coated with gelatin and dried overnight at 37 °C, then dehydrated with ethanol and xylene and mounted with Permount® (Thermo Fisher Scientific, Waltham, USA). Sections were analyzed by using binocular (Leica DMR®) microscope and processed with image J 1.41o® software.

## Theory/calculation

Quantitative analyses were performed in triplo and blinded by quantifying the c-fos positive nuclei in the (unilateral) dorsal horn area of intact 35 μm-thick L6-S1 spinal cord transections. For each individual animal, multiple transections were counted this way and the median number of c-fos positive nuclei per L6-S1 dorsal horn/transection/per animal was calculated. From these medians, the average number of c-fos positive nuclei per L6-S1 dorsal horn/transection/per group was calculated (this will be referred to as “the number of c-fos + cells”). Groups were compared using univariate ANOVA.

## Results

A total of 26 out of 32 individual animals were included in the evaluation. Two animals died prematurely (5 and 15 min before end of distention) during the experiment. In four animals, L6-S1 segments were unfit for evaluation. This diminished sample sizes in the RTX groups (*n* = 3, *n* = 3) and TRPV4 antagonist groups (*n* = 2, *n* = 2). The TRPV4 antagonist groups were omitted from statistical evaluation because of low number of animals. Univariate ANOVA showed statistical significant differences between groups (between-subject effects).

**Fig. 2:**
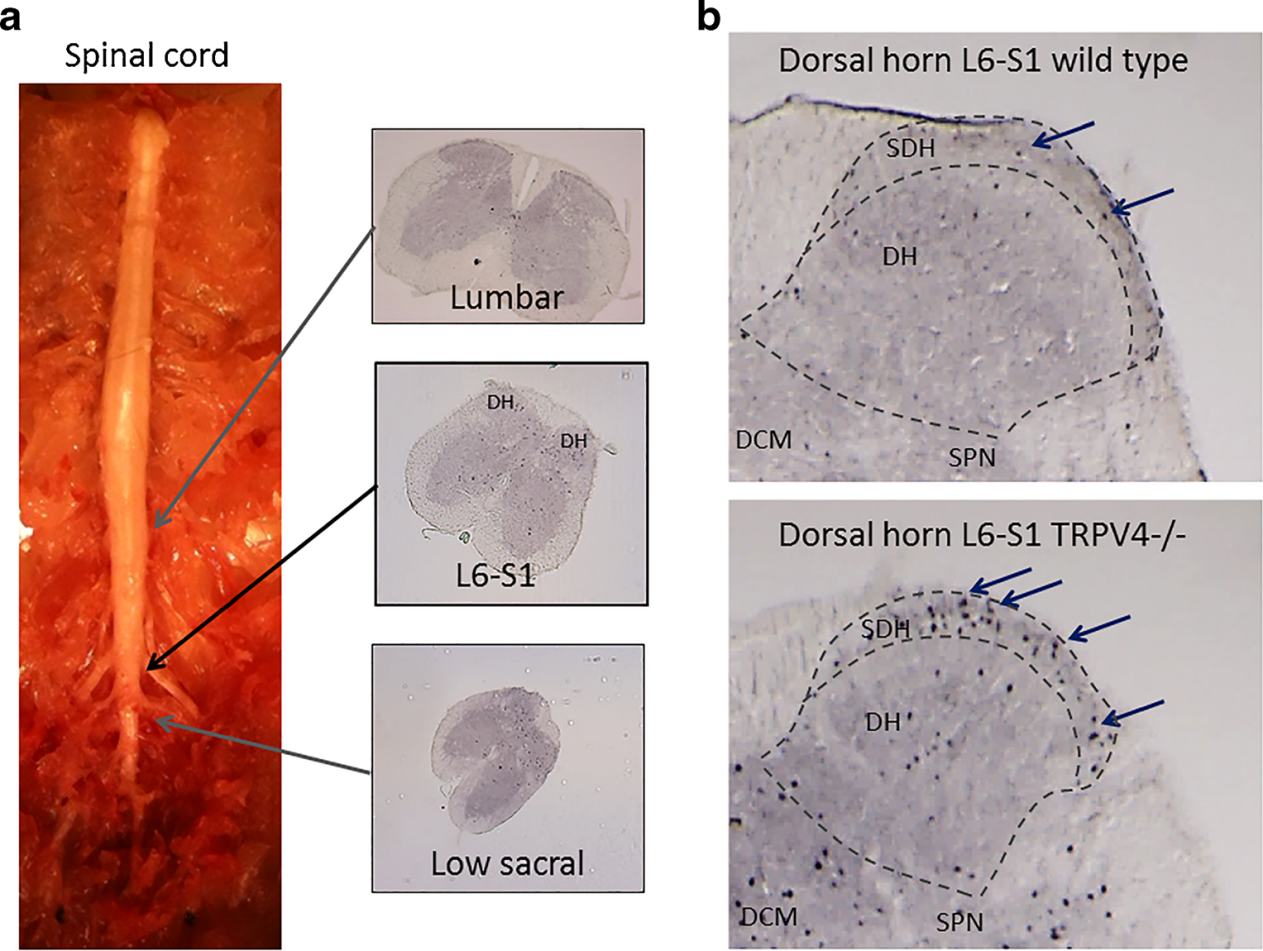
**a** Displays the mouse spinal cord along with the cross sections at different levels showing a distinct differences in size and shape for each level. The dorsal horns (*DH*) of the L6-S1 segment was used for the experiments. **b** Detail of c-fos expression in the dorsal horn (*DH*) area of a L6-S1 spinal cord segments in wild type (*upper image*) and TRPV4 −/− (*lower image*) mouse after bladder distension. C-fos expressing nuclei are seen as *black dots* (*blue arrows*). After bladder stimulation, the majority of c-fos expressing cells in the DH area were seen in the superficial dorsal horn area (*SDH*). Other sensory areas like the sacral parasympathetic nuclei (*SPN*) and the dorsal commissure (*DCM*) also showed increased numbers of c-fos expressing nuclei. TRPV4 −/− mice had more c-fos expressing nuclei after bladder distension compared to wild type mice

Immunohistochemical staining for c-fos were performed on L6-S1 spinal cord segments. Lower sacral and higher lumbar and thoracic spinal cord segments were also dissected and stained and were clearly distinguishable in size and shape from the L6-S1 spinal cord transections (Fig. 2a). IHC experiments showed that only nuclei with c-fos immunoreactivity were detectable and had a clear oval aspect. These c-fos positive nuclei of different sizes were clearly detectable in the designated areas of the spinal cord sections (DH, SPN, DCM) of mice and in rat spinal cord and brain transections with hardly any background (Fig. 2, supplemental Figs. 1 and 2 ). The DAB-nickel immunostaining improved the contrast and detection of c-fos expressing nuclei. The majority of c-fos expressing cells in the dorsal horn region were detected in the superficial dorsal horn area (SDH) followed by the lower (anterior) laminae V area (Fig. 2b). The number of c-fos expressing nuclei in the L6-S1 dorsal horn were counted and compared between groups. In some cases, there were considerable variances observed in the number of c-fos expressing nuclei even between two intact dorsal horns from the same tissue transection. As a result, the experiment used all suitable transections for analysis with an average of six representative L6-S1 transections included for each individual animal.

### Bladder distention

**Fig. 3:**
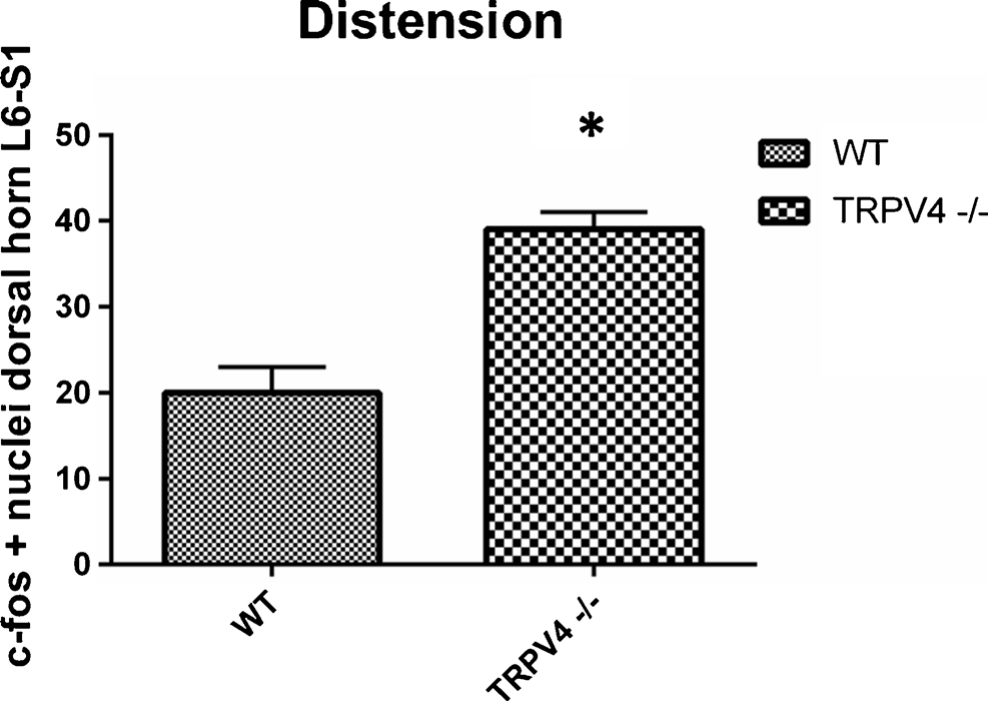
The results of mouse spinal c-fos measurements in L6-S1 dorsal horns after noxious bladder distention (23 cmH2O). C-fos expression was calculated as the average number c-fos expressing cells (nuclei) per L6-S1 dorsal horn/35 μm transection/group. Graph shows that after bladder distention, there was a significant (*) (*p* < 0.001) twofold higher c-fos expression in the L6-S1 dorsal horns of TRPV4 −/− mouse (39 + cells, SD 2) compared to wild type mouse (20 + cells, SD 3) (*p* < 0.001)

**Fig. 4:**
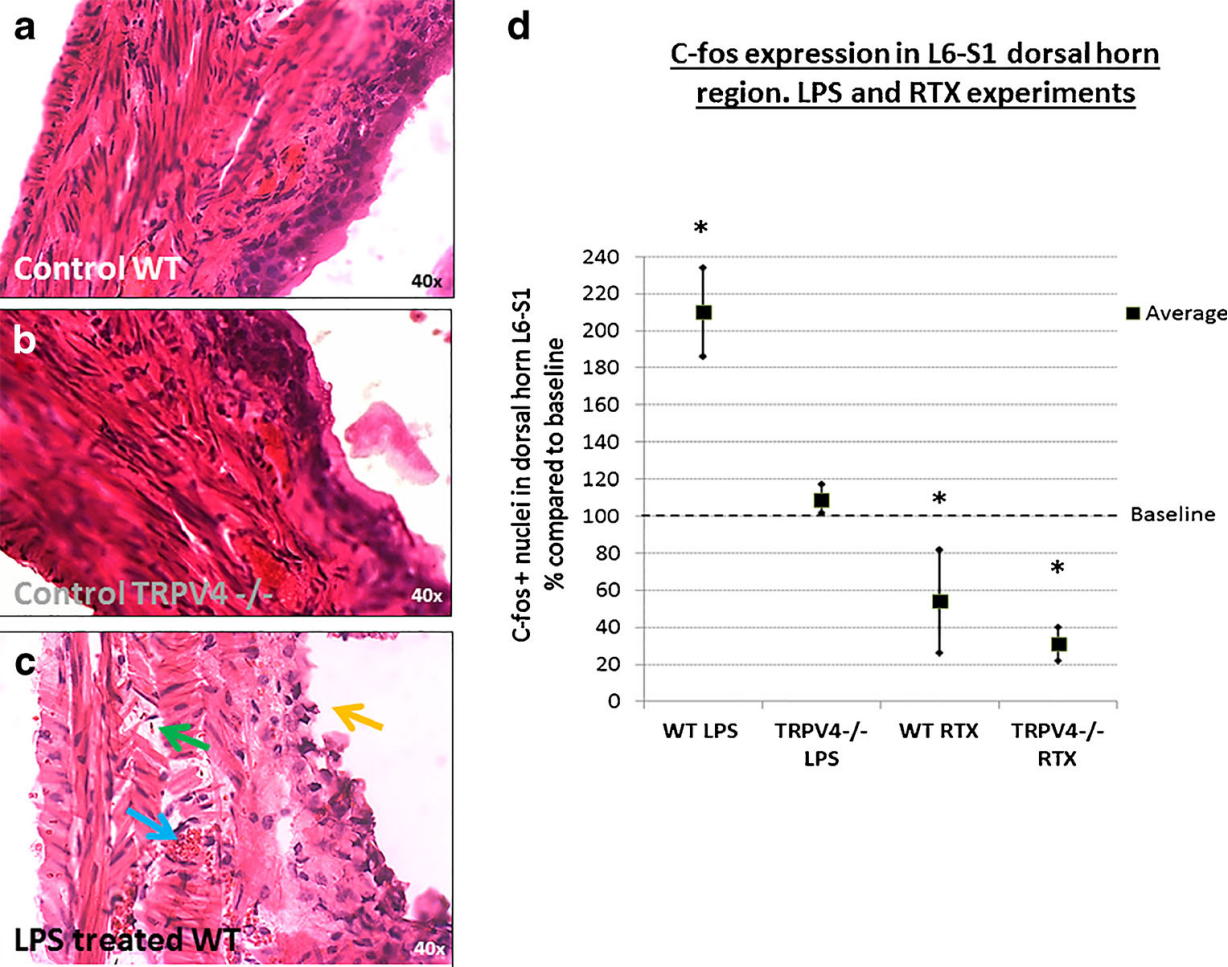
Results of the resiniferatoxin (RTX) and lipopolysaccharide (LPS) experiments. Images **a** and **b** show typical HE staining of WT mouse (**a**) and TRPV4 −/− (**b**) after bladder distention (23 cmH2O). Image **c** shows a WT mouse bladder after LPS treatment + distention. No signs of bladder wall damage or inflammation characteristics were observed in the TRPV4 −/− mice and the WT mice after distention. The LPS treated bladders did show histochemical inflammation characteristics with a disrupted urothelial layer (*orange arrow*), hemorrhage (*blue arrow*), and submucosal edema (*green arrow*) (**c**). Graph **d** shows results of the spinal c-fos quantification from LPS and RTX experiments. TRPV4 −/− and wild type mice received a combination of bladder distention (23 cmH2O) + LPS instillation (bladder inflammation) (WT *n* = 4, TRPV4−/− *n* = 4) or a distention (23 cmH2O) + RTX instillation (desensitizes primary afferent C-fibers) (WT *n* = 3, TRPV4−/− *n* = 3). Treatment groups were compared to bladder distention alone ( = baseline). C-fos expression was calculated as the average number of c-fos expressing cells (nuclei) per L6-S1 dorsal horn/35 μm transection/group. Results are displayed as percent increase or decrease compared to baseline and upper and lower limits display the variation within each group. After LPS treatment, there is a significant (*) 120 % increase in c-fos expression in the wild type mice (42 + cells, SD 5) (*p* < 0.001). By contrast, only a non-significant 17 % increase was seen in the TRPV4 −/− mice after LPS treatment (42 + cells, SD 2). After RTX treatment, c-fos expression was significantly (*) reduced in both wild type (46 %, 11 + cells, SD 2) (*p* = 0.02), and even more profound in TRPV4 −/− mice (69 %, 12 + cells, SD 4) (*p* < 0.001) compared to controls

Distending the bladder with 23 cmH2O resulted in a large difference in c-fos expression between wild type (*n* = 4) and TRPV4 −/− mice (*n* = 4) (Fig. 3). Dorsal horn regions contained a twofold higher number of c-fos positive nuclei in the TRPV4 −/− mouse (39 + cells, SD 2) compared to wild type mouse (20 + cells, SD 3) (*p* < 0.001) (Fig. 3). The bladders were evaluated after the experiment with a standard H&E staining. No signs of inflammation or bladder wall damage after bladder distention were observed in the TRPV4 −/− or the wild types (Fig. 4a, b).

### Bladder inflammation

Inducing bladder inflammation with LPS resulted in inflammatory changes which was evaluated by bladder H&E staining, which revealed a damaged umbrella cell layer, hemorrhage within the bladder wall and bladder wall edema (Fig. 4c, supplemental Fig. 3). Cystitis increased the number of c-fos expressing cells significantly (twofold) in the wild type mice (*n* = 4) (42 + cells, SD 5) compared to controls (*n* = 4) (20 + cells, SD 3) (*p* < 0.001) (Fig. 4d). Induction of cystitis in the TRPV4 −/− mice (*n* = 4) (42 + cells, SD 2), however, did not result in any difference in c-fos expression when comparing them to controls (*n* = 4) (39 + cells, SD 2) (Fig. 4d).

### RTX treatment

Desensitizing the bladder afferent c-fibers with RTX, an ultrapotent TRPV1 agonist, induced a threefold decrease of c-fos activity in the TRPV4 −/− mice (*n* = 3) (12 + cells, SD 4) compared to controls (*n* = 4) (39, SD 2) (*p* < 0.001) (Fig. 4d). In the wild type mouse, a significant decrease in c-fos expression was also seen (*n* = 3) (11 + cells, SD 2) compared to the non-treated controls (*n* = 4) (20 + cells SD 3) (*p* = 0.02) (Fig. 4d). This difference was relatively lower compared to the TRPV4 −/− mice.

### TRPV4 antagonist HC 067047

Fall out in both TRPV4 −/− and wild types resulted in two mice per treatment group. Outcomes of statistics in these low numbers of specimens are therefore problematic and therefore omitted. Results of c-fos activity within each group were very comparable in all groups. A decrease in c-fos expression was observed in the wild type treated group (15 + cells, SD 1) compared to untreated wild types (20 + cells, SD 3). A relatively smaller decrease was also observed in the HC067047 treated TRPV4 −/− group (32 + cells, SD 2) compared to untreated TRPV4 −/− (39 + cells, SD 1).

## Discussion

The spinal c-fos in vivo model was used to investigate the role of TRPV4 channels in bladder afferent signaling during noxious stretch and bladder inflammation. Results of this study demonstrate that the TRPV4 −/− phenotype has disturbed signaling during noxious stimulation of the bladder. Compared to normal mice, there was a twofold higher c-fos expression in the dorsal horns of TRPV4 −/− mice during noxious stretch. This difference was unexpected, since we had hypothesized a decrease of c-fos activity in the TRPV4 −/− mouse. Further experiments were conducted to investigate this afferent signaling paradox. An expected doubling of c-fos expression was seen in normal mice during bladder inflammation. By contrast, no increase in c-fos expression was seen in the TRPV4 −/− mice. The high c-fos counts in the TRPV4 −/− dorsal horns were, however, significantly reduced to low levels by desensitizing primary afferent nerve fibers with the ultrapotent TRPV1 agonist RTX. Our results confirm that TRPV4 is an important mediator in bladder filling sensory pathways, but other receptors of redundant pathways are likely implicated in the TRPV4 −/− mice.

Treatment with the selective TRPV4 antagonist HC 067047 did show homogenous results within each group, but there was considerable fall out of specimens in both treatment groups resulting in small numbers per group (*n* = 2) that were too low for representative statistical analysis. Results are therefore only indicative for future studies. Compared to the controls, the treated wild type group showed a decrease in c-fos expression. This difference was relatively larger compared to the treated TRPV4 −/− mice group. The same antagonist was used previously in bladder in vivo cystometry experiments by Gevaert et al. [11]. Their results demonstrated a significant decrease in the frequency of bladder contractions in the inflamed wild type mouse bladder. Although we used a similar concentration and administrative route in our experiment, we used the TRPV4 −/− antagonist in non-inflamed mouse bladders. The results from this latter study and our own results give an indication that it is worthwhile to further investigate the effects of HC 067047 and other TRPV4 antagonists on bladder afferent signaling pathways.

Spinal c-fos measurement is an established experimental in vivo model for quantifying afferent neural signaling [5]. C-fos is a proto-oncogene that regulates cell differentiation and proliferation [4, 5, 13]. Quantifying afferent neural signals is difficult. The amount of c-fos positive nuclei in designated areas of the spinal cord highly corresponds to the intensity of the nociceptive afferent stimulus and can therefore be used for comparative quantitative analysis [1, 7, 13]. This model has been used for mapping neural pathways and to evaluate the effects of different noxious stimuli and pharmacological intervention on bladder sensory pathways [1, 2, 7]. The only alternative model is electrophysiological recording. There are some differences between these two models. Electrophysiological recordings measure a more direct and continuous afferent signal, while the spinal c-fos model traps a moment in time. Upside of this is that c-fos stains nuclei in specific anatomic areas of the dorsal horns that are very suited for neural mapping, quantification and statistical analysis [13]. Other areas with increased c-fos expression like the sacral parasympathetic nucleus and the dorsal commissure were also detected in our experiments. These areas were not used in our evaluation because isolating these two areas anatomically is more arbitrary compared to the dorsal horn.

A drawback of the c-fos model is the relatively high threshold for detection, since it requires a noxious stimulus for a prolonged time to achieve high enough c-fos concentrations for detection on immunohistochemistry [13]. A stimulus of just over 20cmH2O during 1 h was chosen for two reasons. The first is the requirement of a noxious, but non-traumatic stimulus for spinal c-fos expression. The second was because other groups have demonstrated that a stimulation durance of 1–2 h gives the highest number of c-fos expressing nuclei [1]. Urethane anesthesia was chosen because unlike other forms of anesthesia it does not alter spinal c-fos expression [1]. Both the c-fos model and electrophysiological recordings have great additional value in relation to organ confined measurements like bladder cystometry for understanding the complete afferent pathway of a sensory receptor.

The spinal c-fos model has helped to understand neurologic bladder dysfunction due to spinal cord lesions and was used to investigate the function of sensory receptors like TRPV1 channels and P2X receptors in the urinary bladder [2, 7, 16]. TRPV1 channels are located on bladder afferents and urothelial cells and are involved in pain and inflammatory sensory pathways. The TRPV1 −/− mouse phenotype shows mild voiding abnormalities under normal conditions and shows a decreased spinal c-fos activity during bladder distention and inflammatory conditions [2, 7]. In contrast, the TRPV4 −/− mice phenotype is characterized by an abnormal voiding behavior and a reduced voiding contraction frequency under normal conditions [11]. This suggests that TRPV4 channels are important for normal bladder filling sensations that are largely dependent on low threshold (Aδ) nerve fibers [28]. We expected lower c-fos activity during bladder distention in these knockout mice. Results of this study revealed the opposite meaning that the number c-fos expressing nuclei was equivalent to cystitis conditions in the wild type mouse. During the bladder distention in our experiments, mice were still able to void. As a result, the TRPV4 −/− mice may have received more noxious input during the experiments then the wild type mice, because their bladders maybe responded less to filling and had delayed voiding reflexes and therefore received prolonged periods of bladder wall stretch. This would give some explanation for higher c-fos activity in the knockout. However, it is unlikely that this would fully explain for the very large difference in c-fos expression which were observed in this study. The TRPV4 −/− also responded different to the manipulation of noxious c-fibers compared to the wild types. The LPS experiments demonstrated that bladder inflammation did not cause an expected further rise in c-fos activity in the knockout mice and the RTX instillations decreased the high baseline c-fos expression to a degree that was comparable wild type animals. This relative reduction in c-fos activity was far greater in the TRPV4 −/− mice. RTX is a super analog of capsaicin and targets and incapacitates specifically the TRPV1 expressing noxious c-fibers and does not affect the low threshold Aδ fibers that are responsible for normal bladder filling sensations [8]. Although the experiments cannot differentiate if the remaining c-fos expression after the RTX treatment originates from residual c-fiber signaling or from Aδ signaling. By evaluating the results from the RTX and LPS experiments, we can still discriminate that bladder filling signaling in the TRPV4 −/− mouse is different compared to wild types and is primarily mediated through the noxious, high threshold nerve fibers that express TRPV1 receptors.

The TRPV4 −/− mouse phenotype is remarkable in that it has severe bladder dysfunction with increased micturition intervals, soiling, but normal voiding contractions [11]. TRPV4 channels are located on urothelial cells and upon activation by mechanical stretch trigger the urothelial ATP signaling pathway [24]. Activation of urothelial TRPV4 channels by mechanical stretch most likely occurs through an interaction between TRPV4 channels and a network with the actin cytoskeleton and adherence junctions [12, 14, 22].

**Fig. 5:**
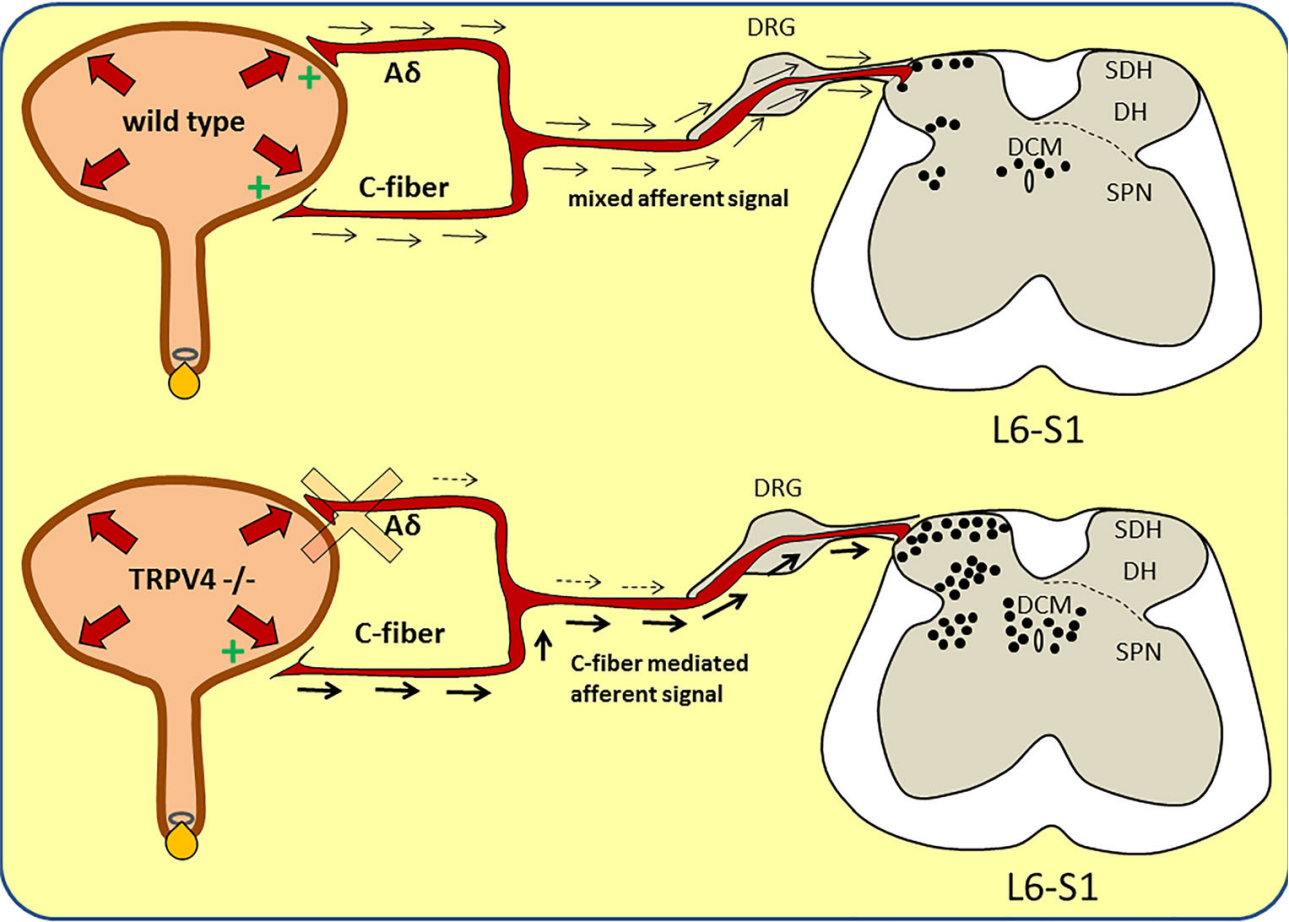
Describing the proposed dysfunctional bladder afferent signaling in the TRPV4 −/− mice. Upper figure shows normal afferent signaling during noxious bladder distention in the wild type mouse and the resulting c-fos expression in the sensory regions: dorsal horns (*DH*; including superficial dorsal horn (*SDH*) areas), sacral parasympathetic nuclei (*SPN*), and the dorsal commissure (*DCM*). Both normal low threshold Aδ (normal bladder filling sensation) and high threshold (noxious) C-fiber afferent nerves are activated during bladder distension giving a mixed afferent signal through the sacral nerves into the sensory areas of the L6-S1 spinal region. C-fos is activated only through noxious stimuli from high threshold nerve fibers, leading to a normal rise in c-fos expression. The lower figure displays the proposed altered bladder afferent signaling in the TRPV4 −/− mice. Based on our results, we suggest that normal low threshold Aδ nerve signaling for normal bladder mechanoreception (filling sensation) is (partly) impaired and results in a compensatory stronger afferent signal through high threshold noxious C-nerve fibers. As a result, more c-fos (twofold) is expressed in the designated sensory areas of the L6-S1 spinal segment

To date, four different types of afferent nerve fibers that are involved in bladder mechanosensation have been identified. Some of these are located in the bladder mucosal area and are be part of the urothelial afferent signaling pathway [6]. The TRPV4 knockout phenotype has an abnormal lower urinary tract function and has altered responses to noxious stimuli in the bladder. This is most likely caused by an adaptation to a congenital impairment of low threshold nerve fiber signaling (Fig. 5). This has to be taken into account when investigating the function of TRPV4 channels in knockout models.

## Conclusions

TRPV4 channels in the bladder are involved in bladder afferent signaling. Bladder filling signaling function in the TRPV4 −/− mice phenotype is more dependent on noxious, high threshold nerves that express TRPV1 receptors.

## Electronic supplementary material

Fig S1 (DOCX 2712 kb)

Fig S2 (DOCX 2523 kb)

Fig S3 (DOCX 4128 kb)

Fig S4 (DOCX 2190 kb)
