# Supplementary material for: TRPV4 mediates afferent pathways in the urinary bladder. A spinal c-fos study showing TRPV1 related adaptations in the TRPV4 knockout mouse
Source: Pflugers Arch. 2016 Aug 5;468(10):1741–9. doi: 10.1007/s00424-016-1859-9 (PMC5026715; doi:10.1007/s00424-016-1859-9)
Supplement: Supplementary file 1 — (DOCX 2712 kb) [file 424_2016_1859_MOESM1_ESM.docx]

**Supplemental data. M&M , figures en legends .**

**Supplemental figure 1**


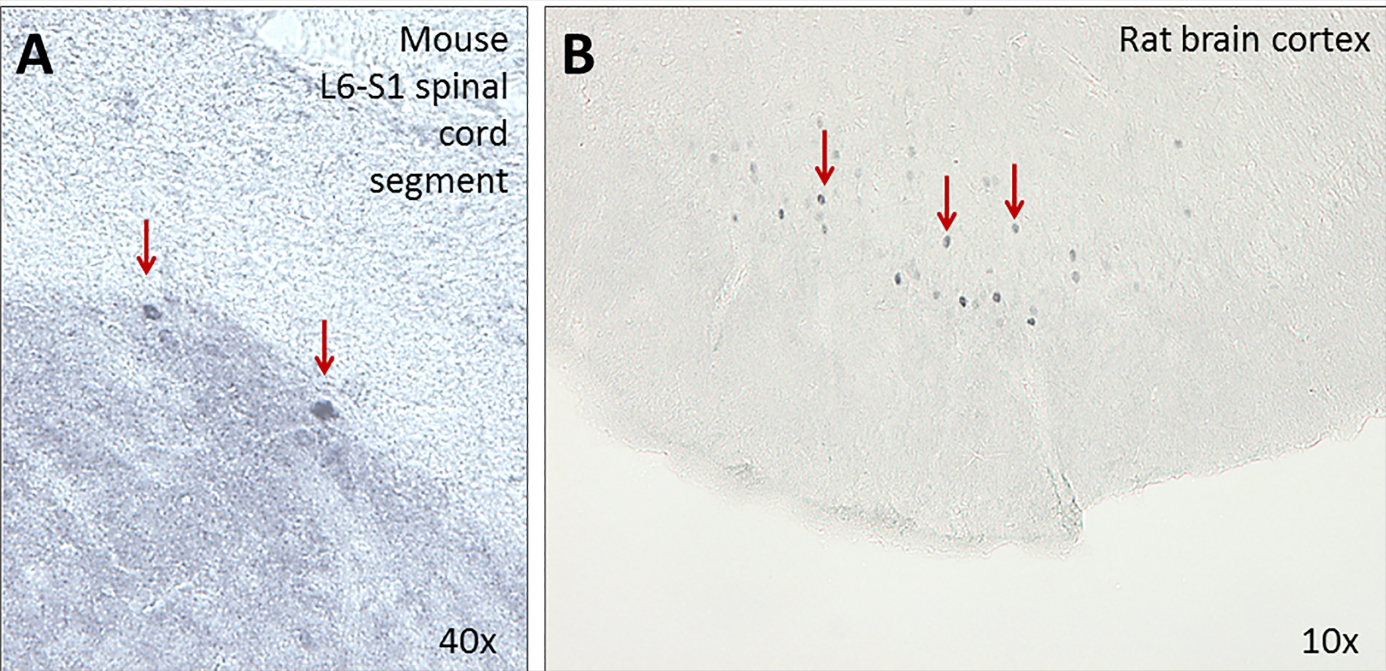


**Fig. 1. Detail of c-fos IHC stainings of a mouse spinal cord and rat brain cortex transection.** Red arrows show oval nuclei that express c-fos, which are clearly distinguishable from background staining.
