# Supplementary material for: TRPV4 mediates afferent pathways in the urinary bladder. A spinal c-fos study showing TRPV1 related adaptations in the TRPV4 knockout mouse
Source: Pflugers Arch. 2016 Aug 5;468(10):1741–9. doi: 10.1007/s00424-016-1859-9 (PMC5026715; doi:10.1007/s00424-016-1859-9)
Supplement: Supplementary file 2 — (DOCX 2523 kb) [file 424_2016_1859_MOESM2_ESM.docx]

**Supplemental figure 2**


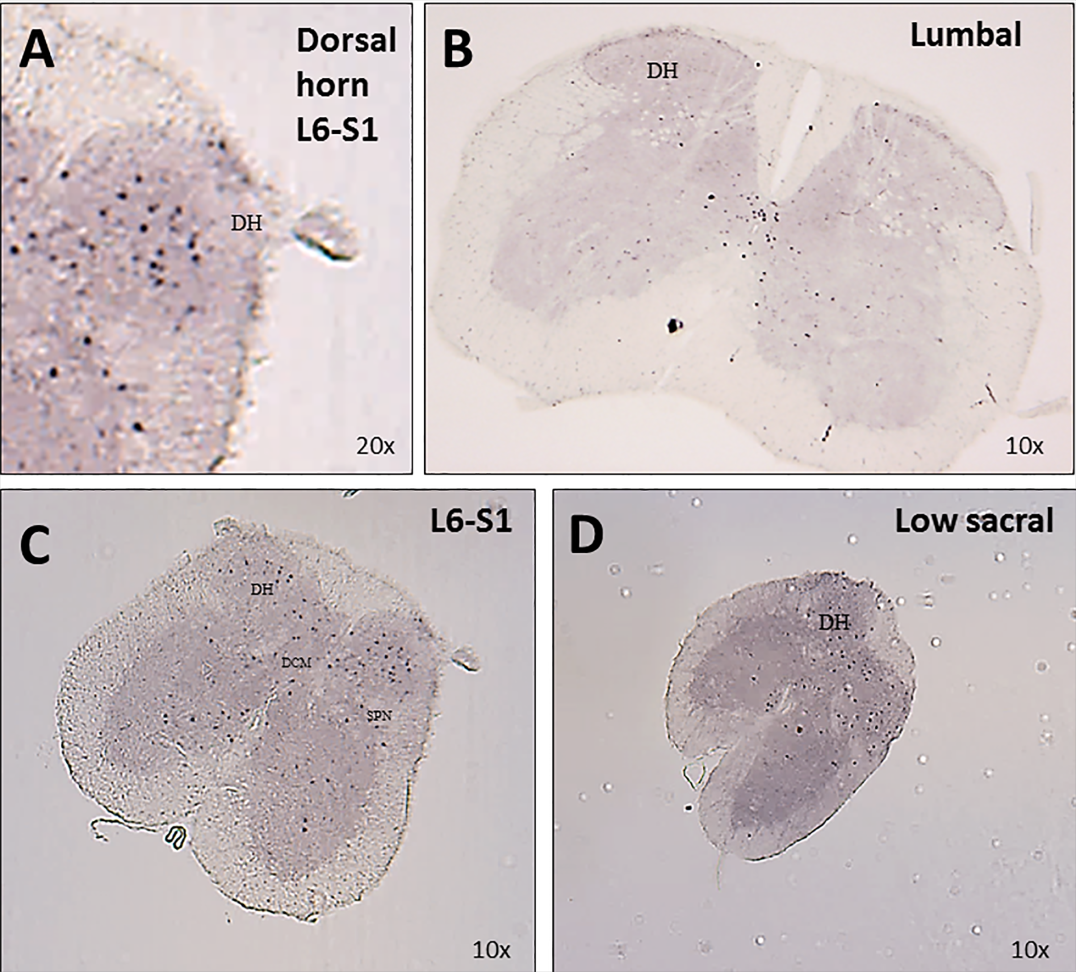


Fig.2 . IHC staining for c-fos on mouse spinal cord transections (35mu). Black-purple oval dots c-fos expressing nuclei. Image A-D show spinal cord transections of a TRPV4 -/- mouse. Image A shows a detail of a dorsal horn of a L6-S1 spinal cord transaction with a large number of c-fos expressing nuclei. Image B shows a lumbal transection with predominantly increased c-fos expression around the dorsal commissure (DCM) and some in the ventral motor horns. Image C is the complete image of the spinal cord transection of image A and shows c-fos expression in the dorsal horns, the DCM and the sacral parasympathetic nuclei (SPN). There is also some c-fos expression in the ventral horns (VH). Image C shows a spinal cord transaction of the smaller low sacral area. Note the size difference between the lumbal , L6-S1 and low sacral spinal cord transections. Also in this area, which is close to L6-S1 and receives afferent information from the pudendal nerve shows high c-fos expression in the dorsal horns and also in SPN , DCM and VH.
