# Supplementary material for: TRPV4 mediates afferent pathways in the urinary bladder. A spinal c-fos study showing TRPV1 related adaptations in the TRPV4 knockout mouse
Source: Pflugers Arch. 2016 Aug 5;468(10):1741–9. doi: 10.1007/s00424-016-1859-9 (PMC5026715; doi:10.1007/s00424-016-1859-9)
Supplement: Supplementary file 3 — (DOCX 4128 kb) [file 424_2016_1859_MOESM3_ESM.docx]

**Supplemental figure 3**


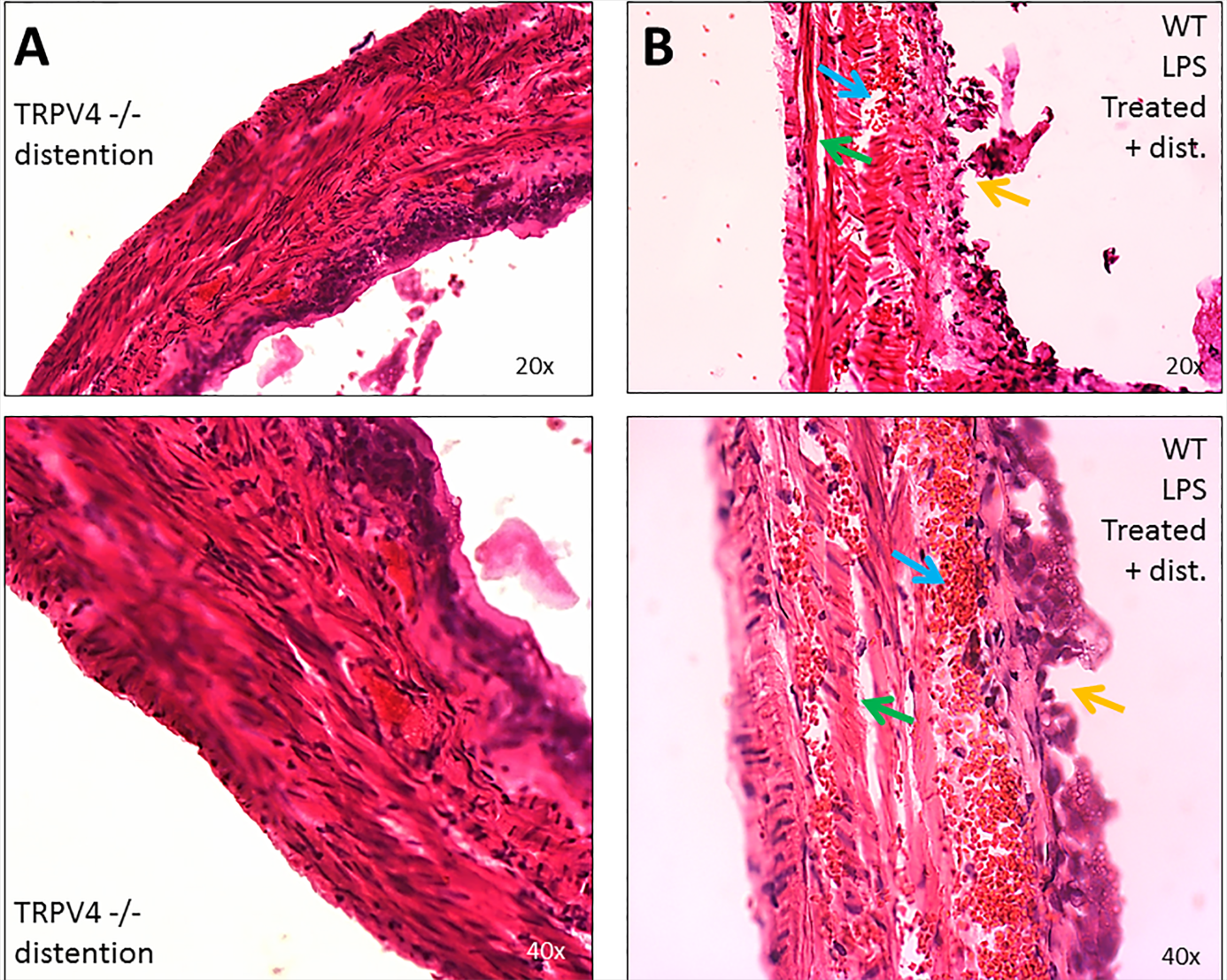


Fig 3. H&E staining of mouse bladders after receiving bladder distention or bladder distention + LPS treatment. Image A (top and bottom) show a TRPV4 -/- mouse bladder with no signs of trauma or inflammation (e.g. intact urothelium, no edema or hemorrhage (red blood cells). Image B shows a wild type bladder that received a LPS treatment prior to a bladder distention. Signa of inflammation are clearly visible with the yellow arrow shows a damaged urothelium, the blue arrow shows hemorrhage with extravasation of red blood cells. The green arrows shows edema.
