# Supplementary material for: TRPV4 mediates afferent pathways in the urinary bladder. A spinal c-fos study showing TRPV1 related adaptations in the TRPV4 knockout mouse
Source: Pflugers Arch. 2016 Aug 5;468(10):1741–9. doi: 10.1007/s00424-016-1859-9 (PMC5026715; doi:10.1007/s00424-016-1859-9)
Supplement: Supplementary file 4 — (DOCX 2190 kb) [file 424_2016_1859_MOESM4_ESM.docx]

**Supplemental figure 4**


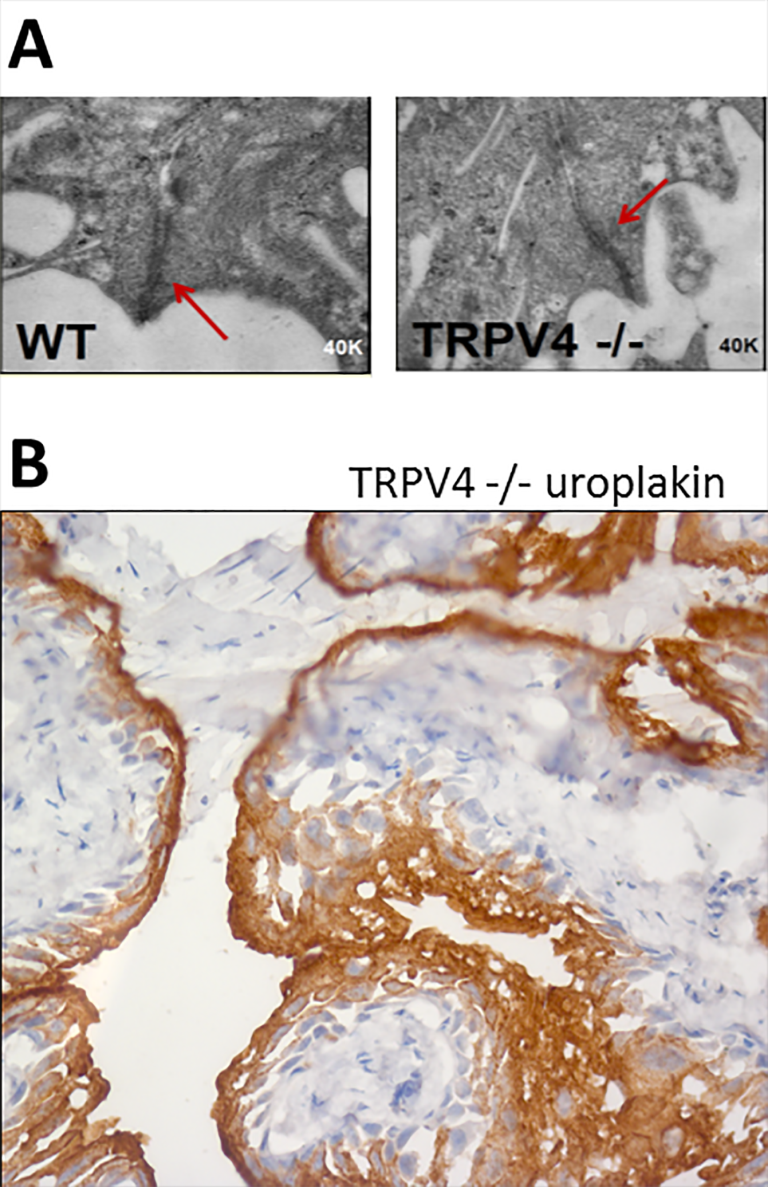


**Fig 4. TEM evaluation and uroplakin III IHC staining in the TRPV4 -/- mouse and wild type mouse to evaluate if essential barrier structures are still intact in the TRPV4 -/-.** Image A shows ultrastructural images taken with a transmission electron microscopy (see M&M below) to show that TRPV4 -/- mice still form tight junctions. Image B is a IHC staining of a TRPV4 -/- bladder using a uroplakin III antibody and shows that uroplakin III is still expressed by the TRPV4 -/- mouse urothelium (also see M&M section below).

Additional M&M for supplemental figure 4.

*Immunohistochemistry*

Tissues were embedded in Tissue-Tek® O.C.T. (Sakura Finetek) and snap frozen in isopentane. From frozen tissue, 4 µm sections were cut in a cryostat microtome (Microm) and air dried. Sections were fixed with 3% v/v paraformaldehyde (10 min), following rinsing (3 times) with Tris-Buffered Saline with 0,05% v/v Tween solution (TBS-T) and blocking with 20% v/v goat-serum in TBS-T. After this, sections were incubated overnight (4°C) with primary antibodies against Uroplakin III (AU1) (Progen Biotecknik GmbH, Heidelberg, Germany). Omission of the first antibody was taken as a control. Poly-HRP-α-RA/Rb/Mo IgG (Immunologic) was used as secondary antibody and bound antibodies were visualized by DAB (Immunologic). Sections were analyzed by using normal binocular microscope (Leica DMR®). Imaged were processed with Image J 1.41o software.

*Transmission electron microscopy (TEM)*

TEM was used to investigate tight junction formation in the wild type and TRPV4 -/- mouse on an ultrastructural level. For this, bladders from wild type and TRPV4 -/- mice were removed. Tissues were fixed in 2% (v/v) glutaraldehyde in 0.1 m phosphate buffer (pH 7.4) for 12 h at 4°C, and post fixed with 1% (w/v) osmium tetroxide in 0.1 m phosphate buffer (pH 7.4) for 1 h. After a rinsing period of 3 h with 0.1 m phosphate buffer (pH 7.4), the samples were dehydrated in an ascending series of ethanols and embedded in Epon 812. Ultrathin sections (60 nm) were picked up on formvar-coated grids, post stained with lead citrate and uranyl acetate, and examined in a JEOL 1010 electron microscope.
